# Supplementary figures and images for: Cell Surface SARS-CoV-2 Nucleocapsid Protein Modulates Innate and Adaptive Immunity
Source: Res Sq. 2021 Dec 13:rs.3.rs-1162804. Preprint. [Version 1] doi: 10.21203/rs.3.rs-1162804/v1 (PMC8687477; doi:10.21203/rs.3.rs-1162804/v1)

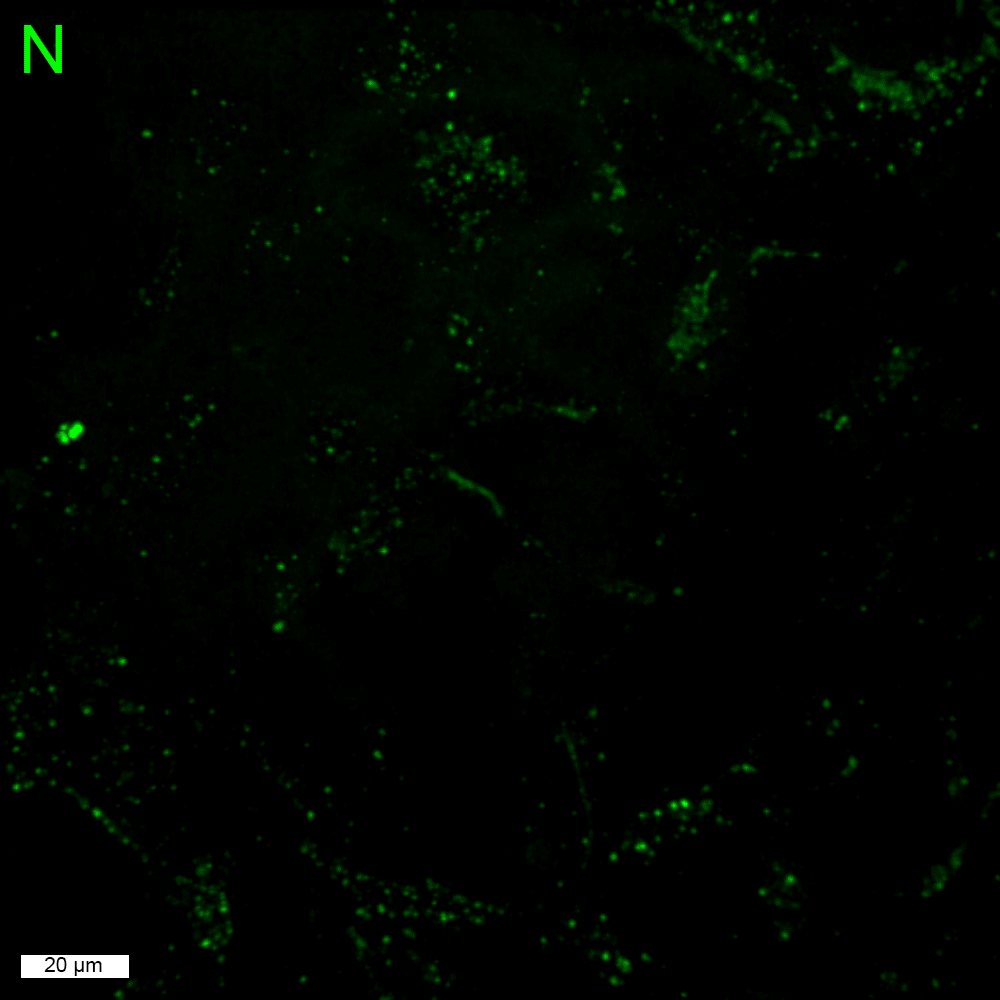

Supplement: Supplement 2 [file 8ec90e03a8ad61fb1bd44d35.gif]

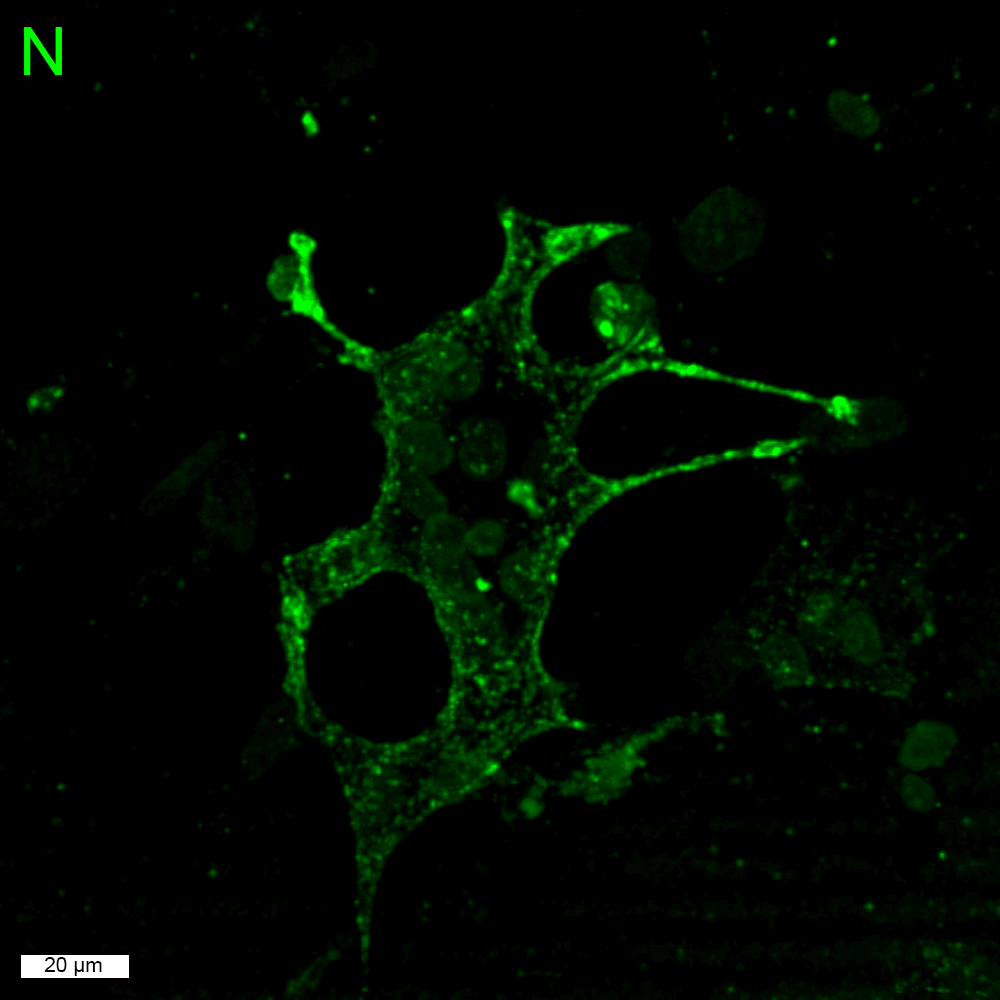

Supplement: Supplement 4 [file 077a48d09757bd8f0eb58184.gif]

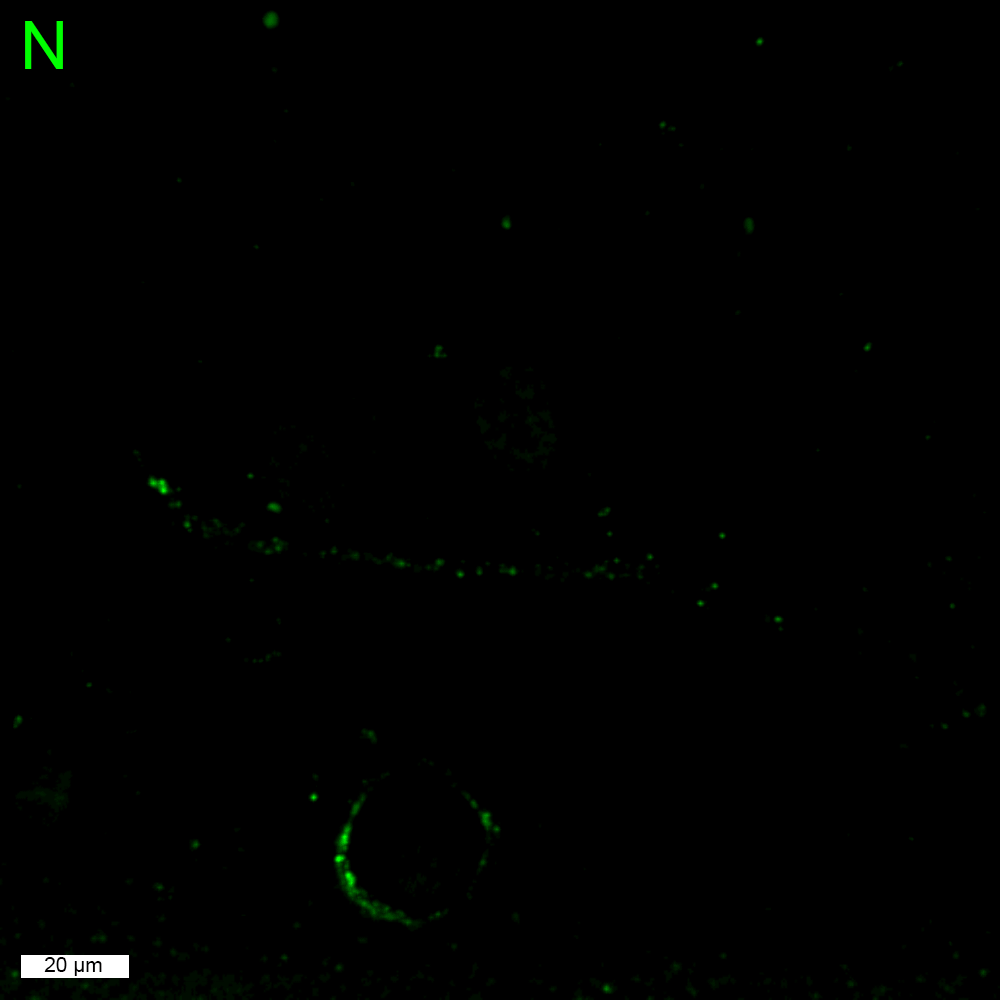

Supplement: Supplement 6 [file 0d5703602a4828f93f7ae0cd.gif]

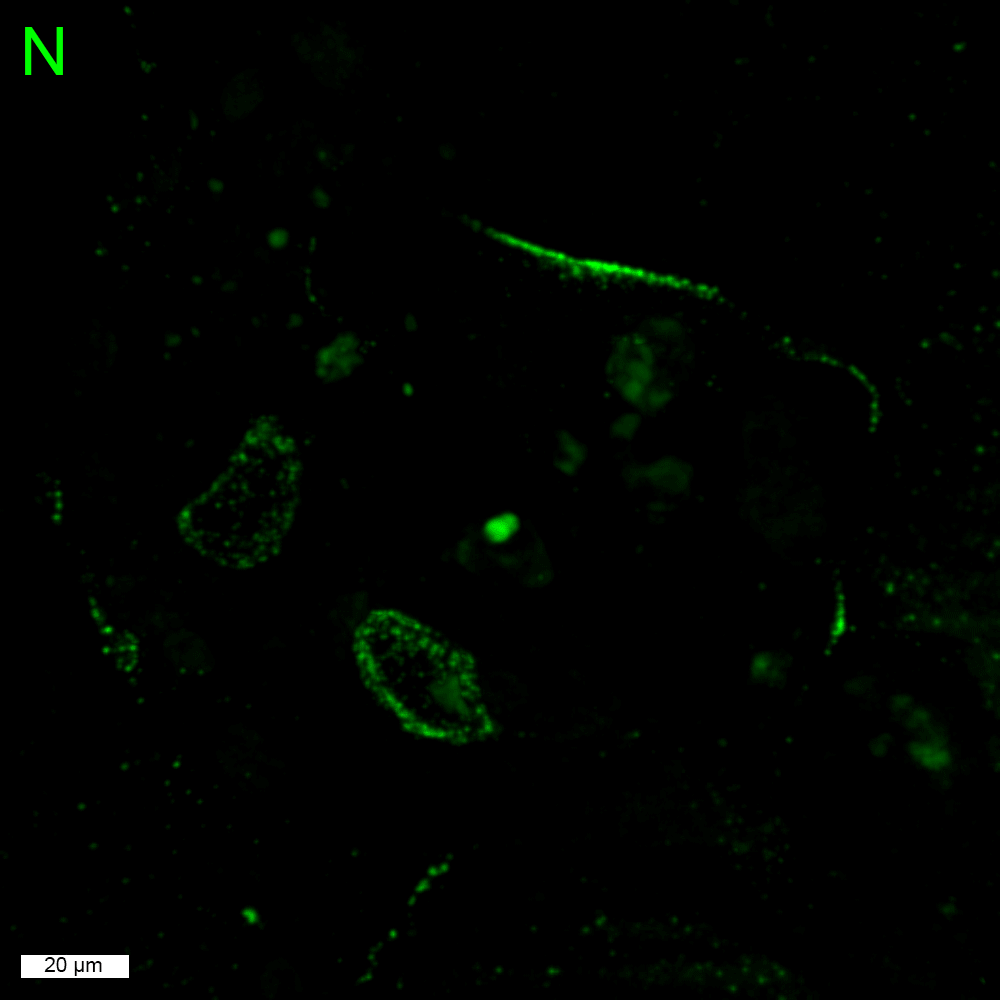

Supplement: Supplement 8 [file 50b83184daa83ff6a81b6172.gif]

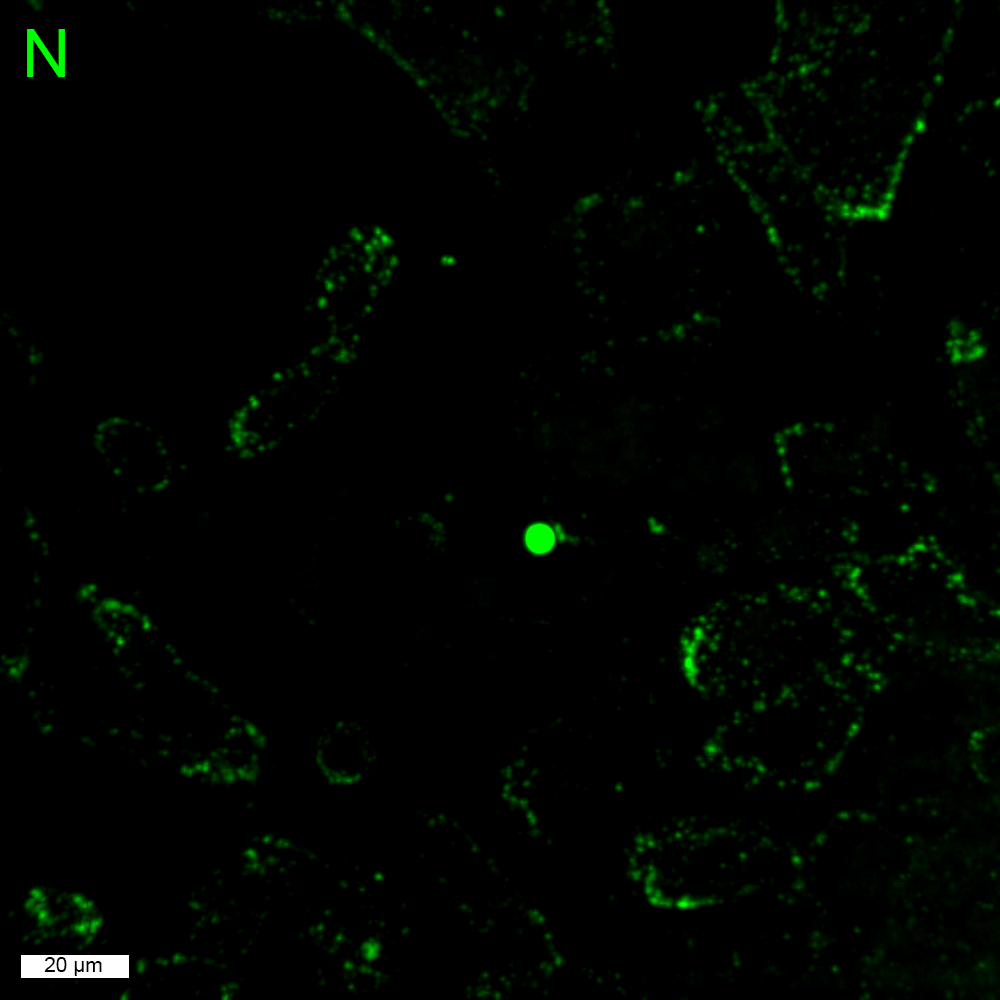

Supplement: Supplement 10 [file 11e7ecf72b6e77783a6d002f.gif]

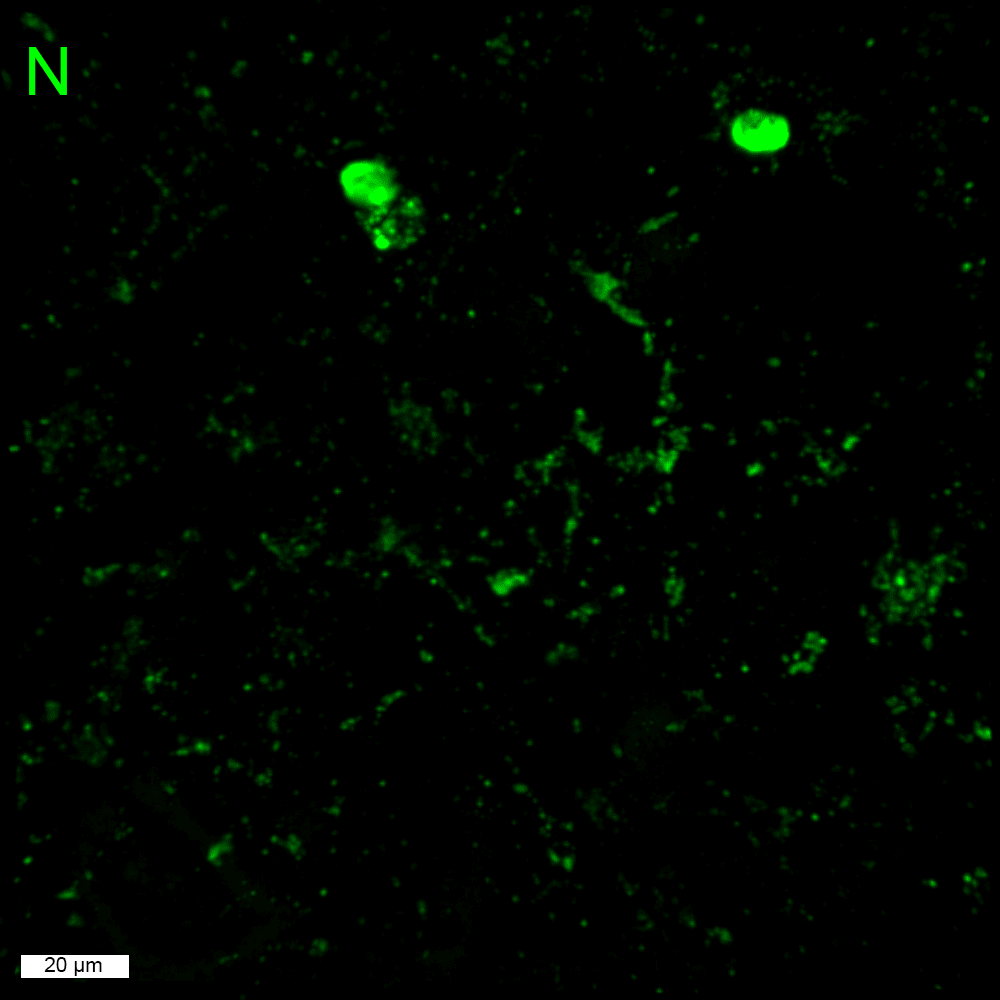

Supplement: Supplement 12 [file 94edfbad65a2b876995024a6.gif]

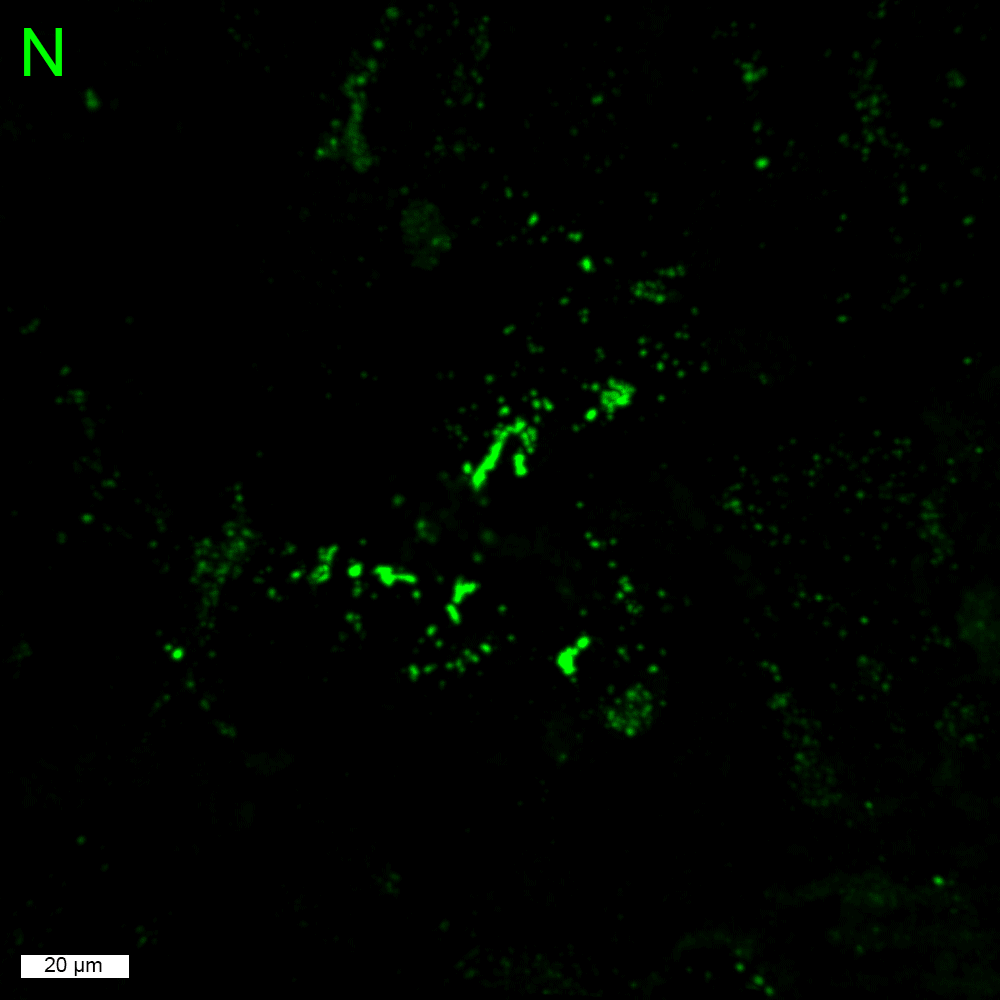

Supplement: Supplement 14 [file d4f77d7858c6990ea5ef2ceb.gif]

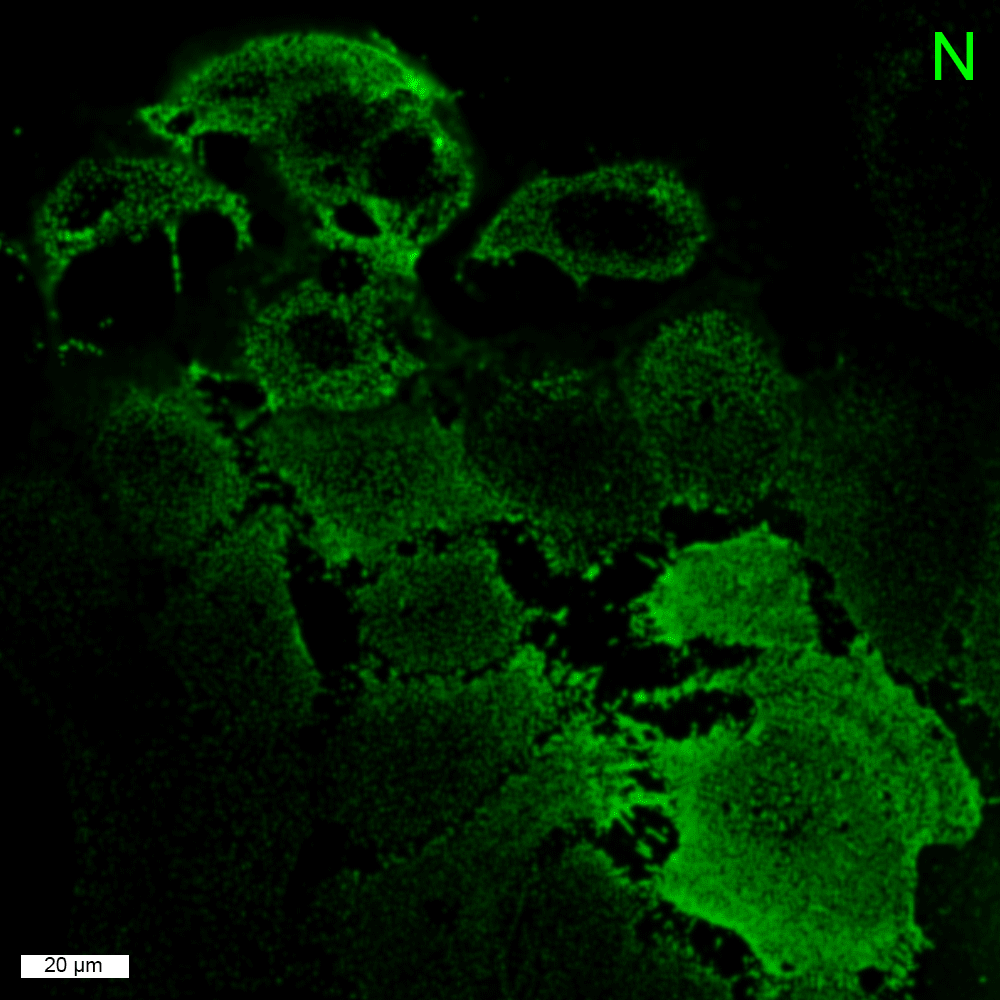

Supplement: Supplement 16 [file d9632ad977c3d494364a3d4f.gif]

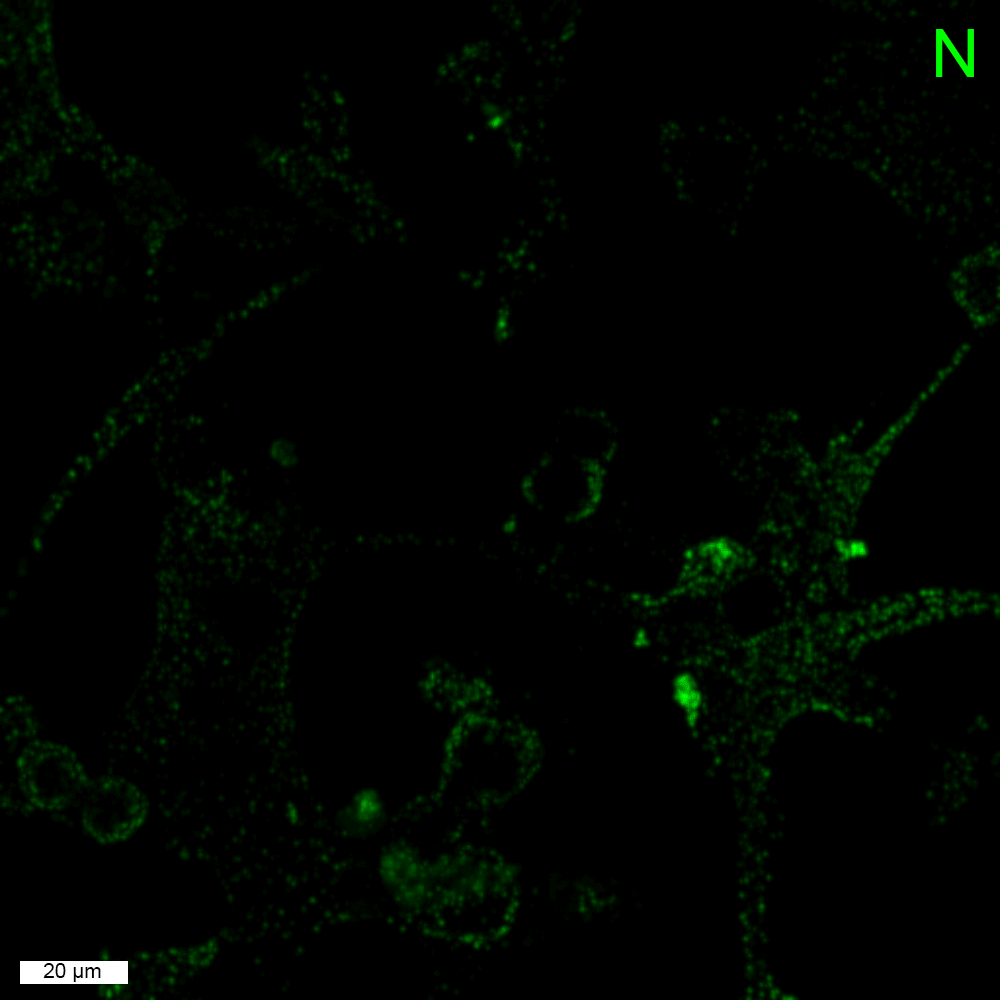

Supplement: Supplement 17 [file d8f97d13db6dc0432816dae2.gif]

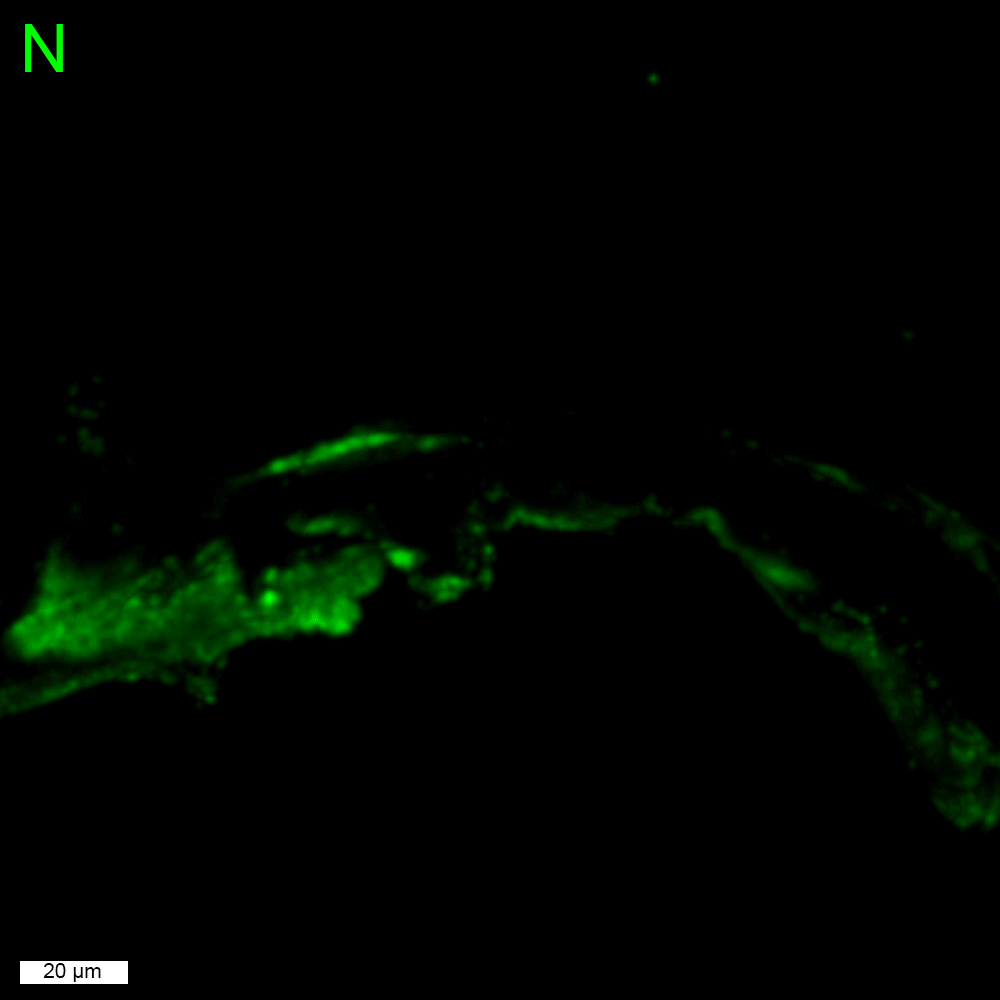

Supplement: Supplement 18 [file 240865a4d31c6186737b1b4c.gif]

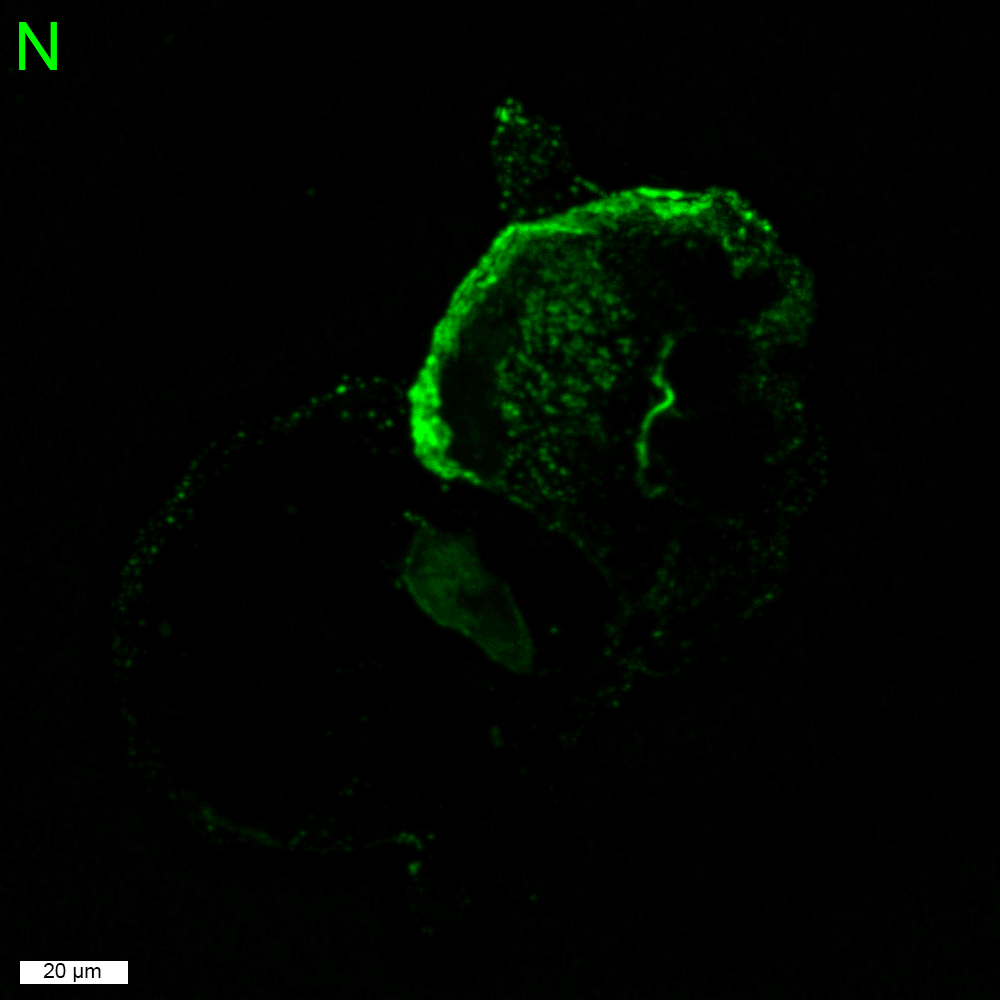

Supplement: Supplement 19 [file dc85425551e88f70299761e8.gif]

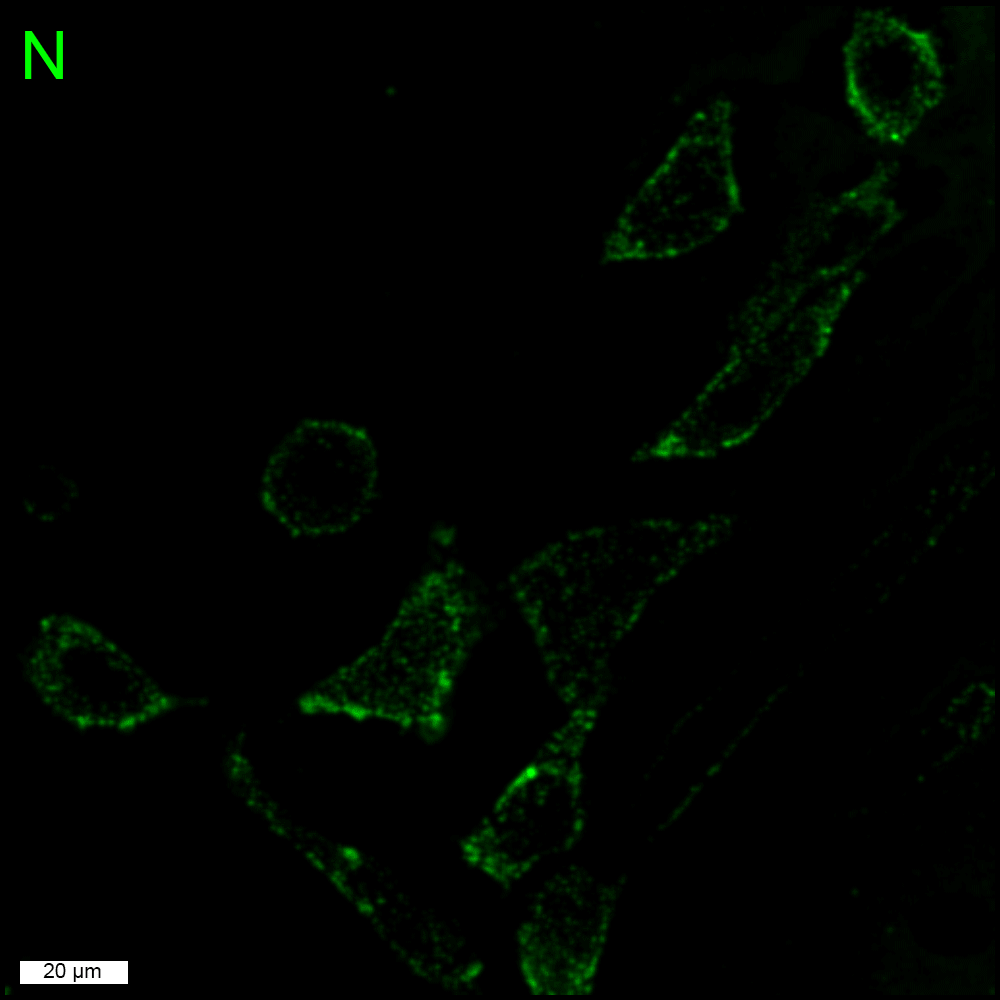

Supplement: Supplement 20 [file 96d3b0bc4d628cf9bc169900.gif]

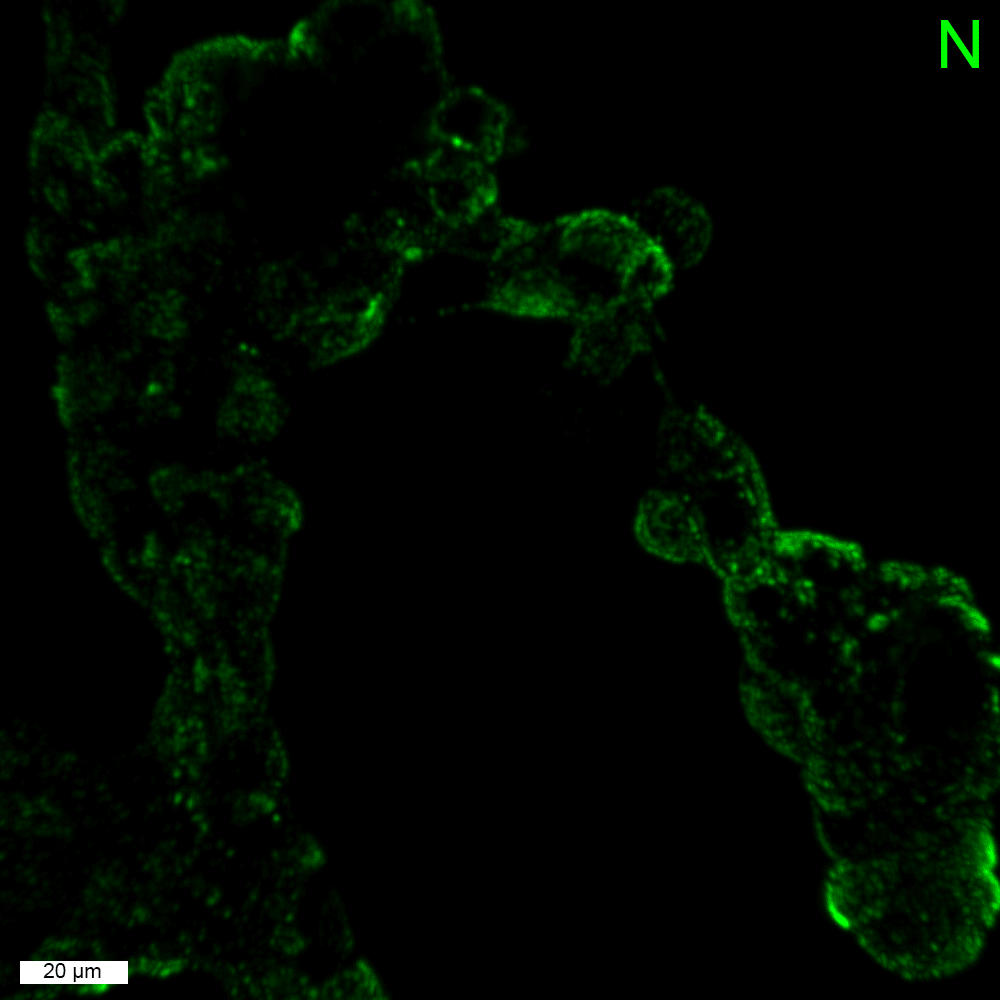

Supplement: Supplement 21 [file fcefb7b6d2965946f1953f40.gif]

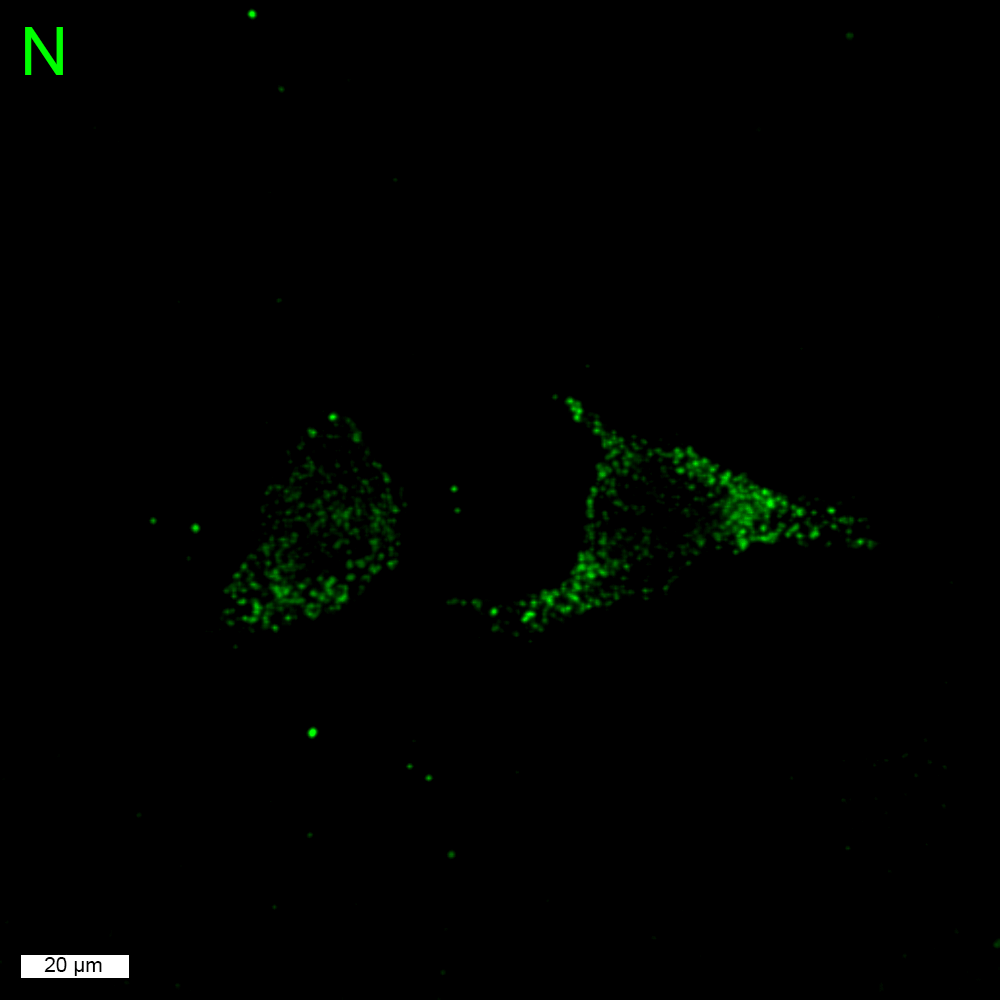

Supplement: Supplement 22 [file 2f4face672cd2d10771cdcb2.gif]
